# The Dynamic Structure of the Estrogen Receptor

**DOI:** 10.4061/2011/812540

**Published:** 2011-07-26

**Authors:** Raj Kumar, Mikhail N. Zakharov, Shagufta H. Khan, Rika Miki, Hyeran Jang, Gianluca Toraldo, Rajan Singh, Shalender Bhasin, Ravi Jasuja

**Affiliations:** ^1^Department of Basic Sciences, The Commonwealth Medical College, Scranton, PA 18510, USA; ^2^Section of Endocrinology, Boston University School of Medicine, Boston, MA 02118, USA; ^3^Medical Genetics Institute, Cedars-Sinai Medical Center, Los Angeles, CA 90048, USA; ^4^Division of Endocrinology and Metabolism, Charles Drew University of Medicine and Science, Los Angeles, CA 90059, USA

## Abstract

The estrogen receptor (ER) mediates most of the biological effects of estrogens at the level of gene regulation by interacting through its site-specific DNA and with other coregulatory proteins. In recent years, new information regarding the dynamic structural nature of ER has emerged. The physiological effects of estrogen are manifested through ER's two isoforms, ER_**α**_ and ER_**β**_. These two isoforms (ER_**α**_ and ER_**β**_) display distinct regions of sequence homology. The three-dimensional structures of the DNA-binding domain (DBD) and ligand-binding domain (LBD) have been solved, whereas no three-dimensional natively folded structure for the ER N-terminal domain (NTD) is available to date. However, insights about the structural and functional correlations regarding the ER NTD have recently emerged. In this paper, we discuss the knowledge about the structural characteristics of the ER in general and how the structural features of the two isoforms differ, and its subsequent role in gene regulation.

## 1. Introduction

**Figure 1:**
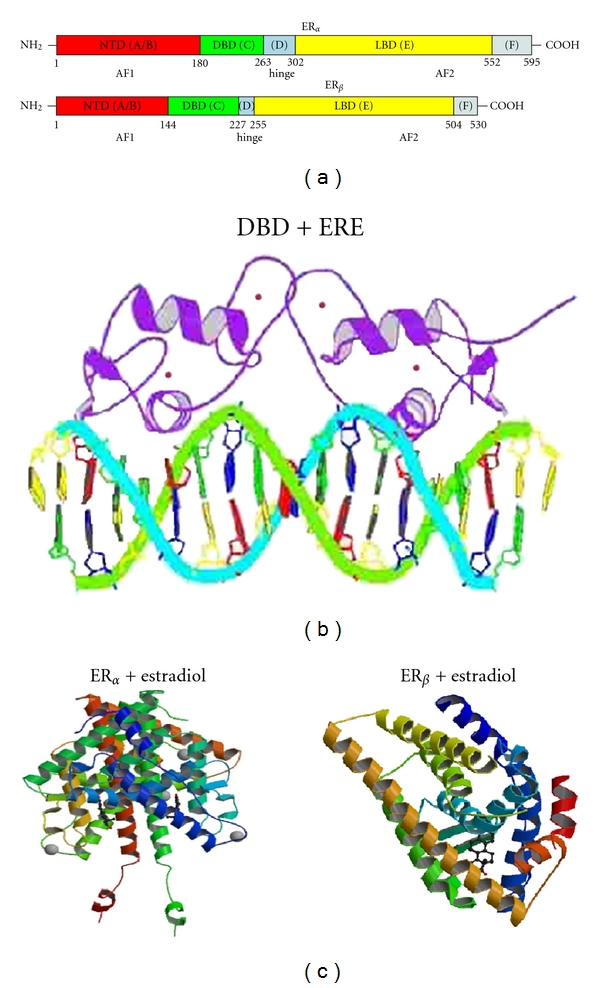
(a) shows the sequence organization of the two isoforms of estrogen receptors, ER_*α*_ and ER_*β*_. Different domains are highlighted in different colors: NTD—amino terminal domain—in red; DBD—DNA binding domain—in green; hinge region—in blue; LBD—ligand-binding domain—in yellow; F region located towards the C-terminal end—in grey. Amino acid sequence position is given for each domain. (b) shows estrogen receptor DBD in complex with DNA-ERE (estrogen response element). Structure 1HCQ from PDB (protein databank) [13]. (c) shows 3-dimensional structures of ER_*α*_ (left) and ER_*β*_ (right) bound to estradiol (PDB structures 1A52 [14] and 3OLS [15]).

The estrogen receptor (ER) is a ligand-inducible intracellular transcription factor that mediates most of the biological effects of estrogens at the level of gene regulation [1–3]. Estrogen biology is exceedingly complex and important in the development and function of numerous tissues and physiological phenomena [4–6]. In the nucleus, the ER up- or downregulates the expression of target genes by interacting through its site-specific DNA and with other coregulatory proteins that include coactivators and corepressors [1–3]. The ligand-bound ER binds as homodimer to specific DNA sequences termed estrogen response elements (EREs) and regulates transcription through interaction with transcription modulators and recruitment of the general transcription machinery [7]. In recent years, new information regarding the ER structures, intra- and intermolecular interactions, posttranslational modifications, and several other factors pertaining to the ER actions has emerged [8–10]. Like other members of the nuclear hormone receptor (NHR) family, the ER is composed of several functional domains that serve specific roles [11]. Starting from NH_2_- to COO-terminus, the principal domains are (1) the N-terminal domain (NTD); (2) DNA-binding domain (DBD); (3) ligand-binding domain (LBD). Two activation function (AF) domains, AF1 and AF2, located within the NTD and LBD, respectively, are responsible for regulating the transcriptional activity of ER [12] (Figure 1(a)).

Full transcription activity of the ER is thought to be achieved by synergism between the two AFs, and their activities are promoter and cell specific [16]. AF1 functions as hormone independent, whereas AF2 function requires the presence of hormone/steroid [12, 17]. In this paper, we focus on the two isoforms of human ER (ER_*α*_ (NR3A1) and ER_*β*_ (NR3A2)), encoded by two different genes. Both have been cloned and characterized [18]. The physiological effects of estrogen are manifested through both ER_*α*_ and ER_*β*_. The ER_*α*_ and ER_*β*_ receptor isoforms display distinct tissue distributions and signaling response [19–21]. ER_*α*_ and ER_*β*_ have also been shown to form hetero dimers on EREs [22]. In terms of sequence homology, the ER_*β*_ shows a high homology to ER_*α*_ in the DBD (more than 95% amino acid identity) and in the LBD (~55% amino acid identity) [19, 22]. However, the NTD of ER_*β*_ is shorter than that of ER_*α*_ with a very poor sequence homology of only *∼*15% compared to that of ER_*α*_. The three-dimensional structures of the independently expressed DBD and LBD have been solved and show overall folds that represent globular proteins with natively ordered conformations [13, 23–25]. To date, no three-dimensional natively folded structure for the NTD is available not only for the ER but for the entire nuclear hormone receptor (NHR) superfamily. Even though the full length structure of the peroxisome proliferator-activated receptor-*γ* (PPAR-*γ*) has been solved, it failed to show any signature of structure formation in its NTD [26]. Warnmark et al. have previously provided insights about the structural and functional correlations regarding the ER NTD [27]. In this paper, we discuss the knowledge about the structural characteristics of the ER and its role in gene regulation.

## 2. The Hinge Region

The “D” domain which follows DBD is known as a hinge region (Figure 1(a)). It contains nuclear localization signal which gets unmasked upon ligand binding and serves as a flexible region connecting DBD and LBD. Hinge regions of ER_*α*_ and ER_*β*_ share only 36% homology [19].

## 3. The “F” Region

The LBD is followed by the C terminal “F” domain, which contains 42 amino acids. Its action was first characterized by Montano et al. by single-point mutations in the domain as well as by whole domain deletion [29]. The “F” domain was found to modulate gene transcription in a ligand-specific manner. The ligand, promoter, and tissue-specific modulation capabilities of the “F” domain were recently studied in detail by Koide et al. [30]. It is also known to impact receptor dimerization [31].

## 4. The Ligand-Binding Domain

Like other NHRs, the “E” domain of ER contains LBD (Figure 1(a)). It consists of 12 helices, contains hormone binding pocket, and is responsible for the most part of functions activated by ligand binding, such as coregulator binding to AF2 [32] and dimerization interface. While ER_*α*_ and ER_*β*_ have both overlapping and unique functions, the overall homology between the ER_*α*_ protein LBD and ER_*β*_ protein LBD does not exceed 55% [19]. However, the two proteins (ER_*α*_ and ER_*β*_) display distinct regions of sequence homology [4, 19]. The amino acid residues 223–343 and 404–457 in ER_*α*_ and ER_*β*_ show a significantly higher homology than that of the sequence encompassing 223–457 and 344–403, respectively [33]. Interestingly, the stretch of the ER LBD amino acid residues 465–468, with lowest homology to ER_*β*_, has been found to be most solvent accessible [34]. On the other hand, the conserved regions with greater homology are protected against degradation and are in direct contact with the ligand [34]. Despite low sequence homology in LBDs within the NHR superfamily, the three-dimensional structural organization of the LBD monomers is strikingly similar. Both isoforms of ER-LBDs have been shown to form dimers with agonist and antagonist ligands. The dimer interface is primarily encompassed by helices 10 and 11.

As a member of the NHR superfamily of transcription factors, ER_*α*_ contains a globular LBD structure that harbors a hormone-binding site, a homo- or heterodimerization interface, and coregulator (activator and repressor) interaction sites [35–38]. The ER_*α*_ LBD structure contains 11 *α*-helices (H1–H12) [24, 39] (Figure 1(c)). The first crystal structure of an ER_*α*_ LBD bound to its natural ligand 17*β*-estradiol (E2) showed that in a compact ellipsoid cavity, E2 is buried in a highly hydrophobic environment [24]. Within this pocket (formed by 22 residues), hydroxyl groups in estradiol at positions 3 and 17 play a crucial role in orienting the steroid/hormone ligand. These hydroxyl groups of the A and D rings are hydrogen bonded to Glu353 from H3, Arg394 from H5, and a water molecule and His524 from H11. In an agonist-bound form, ER_*α*_ is spatially organized in a three-layered structure with helices 4, 5, 6, 8, and 9 lining up on one side by H1 and H3, and on the other side are helices 7, 10, and 11 [24]. Due to the central role of estrogen signaling in diverse diseases ranging from cancer to aging, several synthetic ligands to ER_*α*_ have been developed [40–43]. The crystal structure of the complex of ER_*α*_ LBD bound to the nonsteroidal ligand, diethylstilbestrol, also shows that the hydrophobic interactions primarily govern the accommodation of distinct LBD structures [44]. 

The crystal structures of the human ER_*β*_ bound to genistein [25], estradiol [14] (Figure 1(c)), and rat ER_*β*_ to raloxifene [25] assert the importance of hydrogen bond network on the opposite sides of the respective ligands [45]. The bicyclic moiety of genistein orients in a position similar to the C- and D-ring of E2, facilitating the formation of hydrogen bonds of hydroxyl moieties with histidine groups of the receptor [25]. The specificity of the ligand association between the ER_*α*_ and ER_*β*_ may stem from the distinction in the residues lining the binding pocket [46]. Quite diverse family of compounds (estrogens, some androgens, phytoestrogens, antiestrogens, and environmental estrogens) have been shown in the past to have estrogenizing activity, and to interact with the ER from rat uterus and human breast tumor cells. Interactions of these structurally diverse ligands highlight the intrinsic ER_*α*_ and ER_*β*_ LBD plasticity [47–49].

## 5. The DNA-Binding Domain

Adjacent to the N-terminal transactivation region (A/B domain), a conserved C domain encompasses the DNA-binding sequence [19]. This DNA-binding domain associates with the response elements which can either reside proximally to the promoter regions or enhancer regions located distant from the transcription initiation site [50]. ER DNA binding domain usually binds to the estrogen response element (ERE) composed of a palindromic hexanucleotide 5′AGGTCAnnnTGACCT3′ [51–53]. The DBD of both ER_*α*_ and ER_*β*_ isoforms shares the same DNA response elements. The ERE sequences play an important regulatory role [54, 55]. Not only does it dictate the binding affinity of the ER, but also it has been shown to modulate the recruitment of coactivators [56, 57]. The ER_*α*_ DBD : ERE structures have been studied extensively by several biophysical techniques [13, 23, 55, 58]. Three-dimensional structure of the ER_*α*_ has been solved using nuclear magnetic resonance as well as X-ray crystallographic techniques both alone and in complex with DNA (Figure 1(b)) [13, 23, 55, 58]. The DBD : ERE interactions and ERE-facilitated dimerization are in part mediated through the P box and D box sequences in the Zinc finger domains. These Zn finger subdomains are comprised of 8 cysteine residues that coordinate with the two Zn^+2^ ions. While P box actively interacts with the ERE nucleotides, the D box is present at the dimerization interface [29, 30, 54]. 

The specificity of ER recognition by ERE is exemplified by interesting studies describing its association with glucocorticoid response element (GRE). Three amino acids in the first Zn finger region or ER dictate its interaction with ERE and GRE [13]. Substitution of these three amino acids with the corresponding amino acids from the glucocorticoid receptor's DBD completely changes ER DBD's specificity for an ERE, and it strongly binds to a GRE sequence to initiate GRE-mediated transcriptional activity [13, 23, 54, 55, 58]. Transcriptional regulation at the ERE can be mediated via two separate mechanisms of ER action. Liganded ER can directly associate with specific response element sequences. In the other mode of action, the ER may participate in a multiprotein, preinitiation complex and regulate gene transcription without a direct interaction with any DNA sequence [59–61]. Together, these mechanisms highlight the complex role of coactivators and response elements in eliciting specificity in transcriptional output.

## 6. The N-Terminal Domain

To date relatively little information has been available on the structure of the N-terminal regions of the NHRs. Even though the full-length structure of the peroxisome proliferator-activated receptor-*γ* (PPAR-*γ*) has been solved it failed to show any signature of structure formation in its very short NTD [26]. We and others have shown that the glucocorticoid receptor's N-terminal transactivation AF1 region and a shorter core fragment of AF1, the AF1 core, are unstructured in aqueous solution [62–66]. In other words, the NTD amino acid sequences possess an intrinsically disordered (ID) conformation, a feature of activation domains of many transcription factors [27, 62, 65, 67, 68]. Similar results have been reported for the ER_*α*_ and ER_*β*_, androgen-, and progesterone receptor [69–71]. Thus, activation domains of many signaling proteins including the ER's NTD/AF1 are known to exist in an ID state. One of the reasons for their existence as an ID region seems to be to help them in promoting molecular recognition by providing surfaces capable of binding specific target molecules [72–75]. 

**Figure 2:**
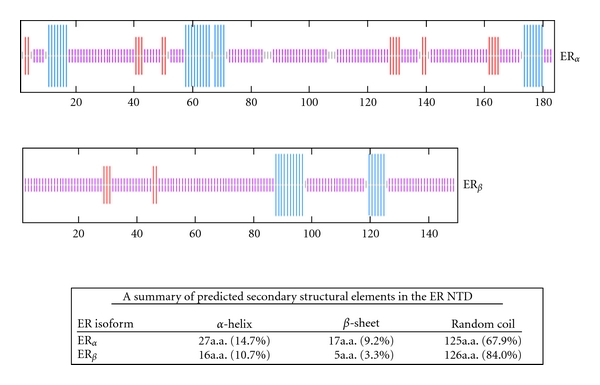
Secondary structural elements predictions of the ER NTD using network protein sequence analysis method as described [28]. Blue, red, and purple colors indicate helix, *β*-sheet, and random coil, respectively. The upper panel: ER_*α*_; the middle panel: ER_*β*_. The table at the bottom summarizes the contents of different secondary structural elements in the NTD of both ERs.

The computational analyses have established that under physiological conditions, the combination of low mean hydrophobicity and relatively high net charge represent an important prerequisite for the lack of well-defined compact structure in proteins or protein regions/domains [75]. The ID nature of the ER NTD/AF1 has been confirmed by circular dichroism method [27]. We performed secondary structural analyses of the ER_*α*_ and ER_*β*_ NTD using network protein sequence analysis [28]. The analytical results show that more than 67% of ER_*α*_ NTD contains random coli conformation, whereas in case of ER_*β*_, the amount of random coil is found to be more than 80% with only a small proportion as helix and sheet in both the cases (Figure 2). It has been proposed that the ID nature of an activation domain allows it to rapidly “sample” its environment until appropriate concentration and affinity of the binding partner proteins are found [65], meaning that they may not be structured until they have recruited and bound their proper interaction partners. Then, either by induced-fit or selective binding of a particular conformer, a high-affinity activation domain : binding partner protein interaction occurs [65, 73]. In case of NHRs' ID NTD/AF1 domains, it has been shown that they undergo a transition to a folded state upon interaction with either components of the general transcription machinery or with other comodulators [76]. 

Several coregulatory proteins are involved in the effect of the ER on target gene transcription. The TATA box-binding protein (TBP) has a central role in the basal transcription machinery and can directly bind to the NTD of the ER_*α*_ but fails to bind to ER_*β*_ NTD and to potentiate ER-activated transcription [27]. This difference in TBP binding could imply differential recruitment of target proteins by the NTDs of ER_*α*_ and ER_*β*_. The affinity of the ER_*α*_ NTD : TBP interaction was determined to be in the micromolar range, as assessed by surface plasmon resonance spectroscopy [27]. Based on these results, it has been proposed that the interaction between ER_*α*_ NTD and TBP may proceed in a two-step manner with initial very fast, low-affinity association, followed by a slow, folding event and tighter association [27]. The initial association may be occurring by electrostatic interactions between the acidic residues of highly negatively charged ER_*α*_ NTD and the positively charged TBP. However, this initial unstable protein complex subsequently may convert into a more stable form by the folding of the ID ER_*α*_ NTD and the formation of specific contacts between the two proteins. In this study, the secondary structures of the independently expressed NTDs of the ER_*α*_ and ER_*β*_ were analyzed using NMR and circular dichroism spectroscopy [27]. 

Secondary structural analyses concluded that both ER_*α*_ and ER_*β*_ NTDs are unstructured in solution [27]. Further, when ER_*α*_ NTD was bound to TBP, structural changes were induced in ER_*α*_ NTD [27]. These results support models of TBP as a target-protein for the N-terminal activation domain of ER_*α*_. Further, the dissociation of this binding suggests a complex behavior, with a rapid dissociation for ER_*α*_ NTD molecules that did not undergo proper folding and a slower dissociation for those molecules that did fold successfully upon physical interaction with the TBP [27]. Such a two-step binding mechanism is consistent with the change in protein conformation that accompanies the ER_*α*_ NTD : TBP interaction. Observed differences in binding of TBP to ER_*α*_ NTD and ER_*β*_ NTD supports a model where the two receptors may be utilizing different sets of target binding proteins [65]. This is consistent with the reports of functional differences between ER_*α*_ NTD and ER_*β*_ NTD where it has been shown that the ER_*α*_ AF1 domain can function in an autonomous manner, whereas the AF1 function of ER_*β*_ cannot [27]. It has also been reported that under most conditions ER_*β*_ possesses a weaker transactivational potency compared to ER_*α*_ [6], and these differences appear to be cell and promoter specific [6]. We have earlier shown that TBP binding induces secondary/tertiary structure formation in the ID AF1 domain of the glucocorticoid receptor such that AF1's interaction with specific coregulatory proteins and subsequent AF1-mediated transcriptional activity is significantly enhanced [77, 78].

Based on the binding of TBP and consequent folding of these ID activation domains, it can be hypothesized that the interaction between NHRs' NTD/AF1 and TBP may be a unified mechanism, through which these ID AF1/NTD acquire a functionally active conformation under physiological conditions. In this conformation, the NTD/AF1 may be able to create favorable protein interaction surfaces for its interaction with specific coregulatory proteins. Of course, the exclusion of certain other binding partners cannot be ruled out. It could thus be hypothesized that a complex and dynamic binding pattern for the N-terminal activation domains of the NHRs occurs to achieve transcriptional activation, where the NTD/AF1 region must be able to obtain different conformations dependent on the binding partner(s). However, a clear picture will emerge only when the functionally folded three-dimensional structure of the NTD/AF1 is solved. At least for now, the differential effects observed in case of two ER isoforms (ER_*α*_ NTD and ER_*β*_ NTD) suggests that TBP may not be a common coregulator that must bind/fold all the NHRs' NTD/AF1. Thus, it is quite possible that other protein components from the basal transcription machinery may provide such interactions. In fact, we and others have observed that at least in case of the androgen receptor, its ID NTD/AF1 undergoes disorder/order transition through its interaction with RAP74, a subunit of TFIIF, an important component of basal transcription machinery [70, 79].

## 7. Summary and Perspectives

Recent observations have led to the conclusion that in cells, ER and several other NHRs behave very dynamically such that their kinetic behavior in cells allows them to rapidly interact with various coregulatory proteins, and with chromatin and DNA [80]. Further, the ER moves to various sites in cells to function, and the local concentrations and various other constellations of potential coregulatory proteins are required to associate with the ER to activate or repress the expression of target genes [80]. The LBD crystal structures have clearly demonstrated that differing sets of coactivators/corepressors come together in response to agonist or antagonist ligand binding, such that agonist in one cell type can be an antagonist in another cell type. The overall picture is one of a complex, dynamic network controlled by the ER. It is not yet clear whether unique tissue/cell-specific coregulatory protein interactions can fully explain the tissue/cell-specific actions of the ER and other NHRs. When the clear picture will emerge, it is certain that other dynamic considerations will prove to be the dominant underlying mechanism.
